# Analysis of Depressive Symptoms and Perceived Impairment Among Physicians Across Intern Year

**DOI:** 10.1001/jamanetworkopen.2021.44919

**Published:** 2022-01-25

**Authors:** Lisa M. Meeks, Jennifer Cleary, Adam Horwitz, Karina Pereira-Lima, Zhuo Zhao, Yu Fang, Srijan Sen

**Affiliations:** 1 Department of Family Medicine, University of Michigan Medical School, Ann Arbor; 2 Center for a Diverse Healthcare Workforce, School of Medicine, University of California, Davis, Sacramento; 3 Eisenberg Family Depression Center, University of Michigan, Ann Arbor; 4 Department of Psychology, University of Michigan, Ann Arbor; 5 Michigan Neuroscience Institute, University of Michigan, Ann Arbor; 6 Department of Psychiatry, University of Michigan Medical School, Ann Arbor

## Abstract

This cohort study compares perceived impairment associated with depressive symptoms among physicians before intern year vs during intern year.

## Introduction

Resident physicians experience high levels of depressive symptoms.^1^ While stigma and career concerns disincentivize help-seeking, this may also be associated with the normalization of depressive symptoms (eg, trouble sleeping, feeling down about oneself) during training, skewing residents’ perception of their functioning.^2^ Perceptions that symptoms are not serious is a commonly reported barrier to treatment even among individuals with suicidal ideation,^3^ yet untreated depression presents clear risks to interns (eg, progression to suicidal ideation) and their patients (eg, increased risk for medical errors).^4^

To our knowledge, no studies have assessed the extent to which perceptions of impairment associated with depressive symptoms change with the start of residency. Thus, we used data from the Intern Health Study to assess changes in associations between depressive symptoms and perceived impairment prior to vs during intern year.

## Methods

This cohort study was approved by the University of Michigan institutional review board and followed the Strengthening the Reporting of Observational Studies in Epidemiology (STROBE) reporting guideline. All participants provided electronic informed consent. Intern Health Study recruitment procedures and study design are detailed in a prior publication.^5^ Preinternship (June) and internship (September or December) depressive symptoms and impairment item data were obtained from the 2007to 2018 cohorts. The Patient Health Questionnaire-9 (PHQ-9) is a validated self-report of depression symptoms corresponding to the *Diagnostic and Statistical Manual of Mental Disorders* (Fourth Edition) (*DSM-IV*) symptoms of clinical depression. Respondents reported symptom frequency over the previous 2 weeks (0 = never to 3 = nearly every day) for a total score of 0 to 27. Depression scores are categorized as minimal (0-4), mild (5-9), moderate (10-14), moderate to severe (15-19), and severe (≥20). A single item corresponding to the *Diagnostic and Statistical Manual of Mental Disorders* (Fifth Edition) (*DSM-5*) clinically significant impairment criterion for major depression asked respondents to report the difficulty of performing social, occupational, and other important activities owing to these symptoms (0 = “have not experienced any depressive symptoms or no difficulty at all,” 1 = “somewhat difficult,” 2 = “very difficult,” and 3 = “extremely difficult”).^6^ Race and ethnicity were self-reported in the questionnaire; available categories were Arab or Middle Eastern (added in 2016), Asian (eg, Indian or Chinese), Black or African American, Latino or Hispanic, multiracial, Native American, Pacific Islander, White, and other. Multiracial was used when respondents chose more than 1 race or ethnicity. Other races and ethnicities were those hand-entered by respondents. This information was assessed to investigate racial and ethnic disparities in training physician mental health.

To characterize associations among PHQ-9 scores, perceived impairment, and internship, we compared mean PHQ-9 scores within impairment categories before vs during internship via *t* tests and descriptive statistics conducted in R statistical software version 3.5.3 (R Project for Statistical Computing). We included assessments from interns with preinternship and 1 follow-up assessment using the next-nearest assessment. All statistical tests were 2-sided using statistical significance cutoffs of *P* < .05. Data were analyzed from January to November 2021.

## Results

Among 15 566 interns who provided data before and during internship, the mean (SD) age was 27.5 (2.7) years and there were 7985 (51.3%) women. There were 3331 Asian individuals (21.4%), 560 Black individuals (3.6%), 545 Latino or Hispanic individuals (3.5%), 1105 multiracial individuals (7.1%), 9495 White individuals (61.0%), and 530 individuals with other race or ethnicity (3.4%).

Mean (SD) PHQ-9 perceived scores associated with each perceived impairment category were significantly increased during internship compared with preinternship (no symptoms or no difficulty: 2.4 (2.4) vs 0.9 (1.4); *t*_20 074_ = 72.33; *P* < .001; somewhat difficult: 4.9 (3.2) vs 3.1 (2.2); *t*_3794_ = 28.98; *P* < .001; very difficult: 9.0 (4.1) vs 6.3 (3.7); *t*_224_ = 10.06; *P* < .001; extremely difficult: 14.2 (4.6) vs 9.4 (5.9); *t*_67_ = 6.57; *P* < .001) (Table). All median (IQR) during-internship measurements within each category were also significantly increased compared with preinternship measurements (no symptoms or no difficulty: 1.0 [0.0-3.0] vs 3.0 [2.0-6.0]; *P* < .001; somewhat difficult: 6.0 [3.0-9.0] vs 9.0 [6.0-12.0]; *P* < .001; very difficult: 9.5 [5.0-13.4] vs 14.0 [11.0-17.0]; *P* < .001; extremely difficult: 5.5 [1.0-15.0] vs 17.0 [14.0-20.8]; *P* < .001) (Figure). The median (IQR) for depressive symptom scores reported as very or extremely difficult prior to internship was similar to the median (IQR) for scores reported as somewhat difficult during internship (9.0 [3.0-14.0] vs 9.0 [6.0-12.0]).

**Table.** PHQ-9 Depressive Symptoms Scores by Impairment Category

Perceived impairment level	PHQ-9 score, mean (SD)		Test statistic, *t*^a^	*df*
	Preinternship	Internship		
No symptoms or no difficulty	0.9 (1.4)	2.4 (2.4)	72.33	20 074
Somewhat difficult	3.1 (2.2)	4.9 (3.2)	28.98	3794
Very difficult	6.3 (3.7)	9.0 (4.1)	10.06	224
Extremely difficult	9.4 (5.9)	14.2 (4.6)	6.57	67

Abbreviations: *df*, degrees of freedom; PHQ-9, Patient Health Questionnaire-9.

^a^ All *P* values < .001.

**Figure.**
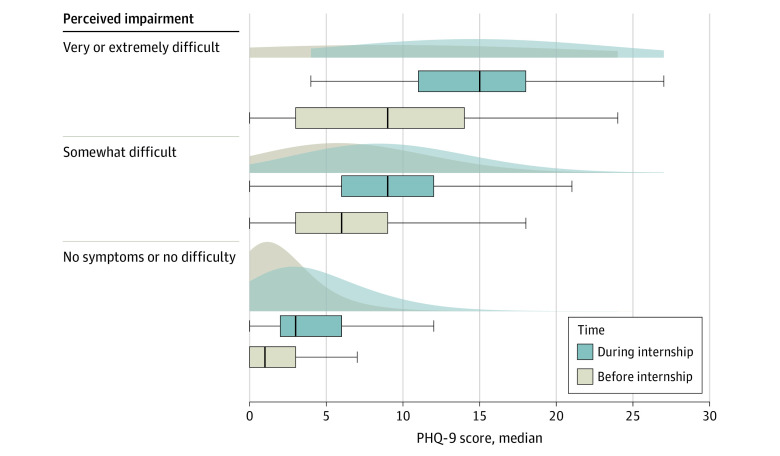
Patient Health Questionnaire-9 (PHQ-9) Scores by Level of Perceived Impairment in Functioning Shaded areas indicate distribution; vertical lines, medians; boxes, IQRs; whiskers, 95% CIs.

## Discussion

This cohort study of more than 15 000 interns found that the mean severity of depressive symptoms that interns viewed as functionally impairing during internship year was increased compared with preinternship. This may be associated with a rising bar, in which symptom severity previously described as very impairing is normalized after entering internship as being somewhat impairing. This potentially suggests a shift in interns' ability to recognize the impact of depressive symptoms^2^ in the context of medical training. Limitations of this study include response biases (eg, fear of reporting any impairment) and nonrandom attrition that may limit generalizability.

Failure to perceive impairment carries implications for interns (eg, lack of help-seeking), training programs (eg, potential attrition of interns), and patient care (eg, disengagement and medical errors). Future research should investigate whether these discrepancies are associated with interns’ ability to effectively cope or with failure to recognize impairment owing to symptom normalization; associations between impairment and help-seeking, including requests for disability accommodation; and whether personalized feedback on depressive symptoms and potential impairment is associated with recalibration of misperceptions. Recognizing impairment may be associated with decreased adverse outcomes, including medical errors^3^ and attempted suicide among interns.
